# Chemically-Induced Inflammation Changes the Number of Nitrergic Nervous Structures in the Muscular Layer of the Porcine Descending Colon

**DOI:** 10.3390/ani11020394

**Published:** 2021-02-04

**Authors:** Liliana Rytel, Ignacy Gonkowski, Waldemar Grzegorzewski, Joanna Wojtkiewicz

**Affiliations:** 1 Department of Internal Disease with Clinic, Faculty of Veterinary Medicine, University of Warmia and Mazury, Street Oczapowskiego 14, 10-719 Olsztyn, Poland; 2 Students’ Scientific Club of Pathophysiologists, Department of Human Physiology and Pathophysiology, School of Medicine, University of Warmia and Mazury, 10-082 Olsztyn, Poland; gon.ign@wp.pl; 3 Interdisciplinary Center for Preclinical and Clinical Research, Department of Biotechnology, Institute of Biology and Biotechnology, College of Natural Sciences, University of Rzeszow, Pigonia 1 str., 35-310 Rzeszow, Poland; wgrzegorzewski@ur.edu.pl; 4 Department of Human Physiology and Pathophysiology, School of Medicine, University of Warmia and Mazury, 10-082 Olsztyn, Poland

**Keywords:** nitric oxide, inflammation, enteric nervous system, descending colon

## Abstract

**Simple Summary:**

The enteric nervous system (ENS) is the part of the nervous system that is located in the wall of the gastrointestinal tract and regulates the majority of the functions of the stomach and intestine. The ENS is characterized by a complex structure and a high degree of independence from the brain. It is known that the ENS changes under the impact of physiological and pathological stimuli. One of the active substances synthetized by enteric neurons is nitric oxide (NO), which is involved in the regulation of intestinal motility, blood flow, secretory activity, and immunological processes in the gastrointestinal tract. In the present study, the influence of chemically-induced inflammatory process on a number of nitrergic neuronal structures located in the muscular layer of the descending colon is investigated. An increase in the number of structures that nitric oxide takes part in is correlated with the inflammatory processes.

**Abstract:**

The enteric nervous system (ENS) is the part of the nervous system that is located in the wall of the gastrointestinal tract and regulates the majority of the functions of the stomach and intestine. Enteric neurons may contain various active substances that act as neuromediators and/or neuromodulators. One of them is a gaseous substance, namely nitric oxide (NO). It is known that NO in the gastrointestinal (GI) tract may possess inhibitory functions; however, many of the aspects connected with the roles of this substance, especially during pathological states, remain not fully understood. An experiment is performed here with 15 pigs divided into 3 groups: C group (without any treatment), C1 group (“sham” operated), and C2 group, in which experimental inflammation was induced. The aim of this study is to investigate the influence of inflammation on nitrergic nervous structures in the muscular layer of the porcine descending colon using an immunofluorescence method. The obtained results show that inflammation causes an increase in the percentage of nitric oxide synthase (nNOS)-positive neurons in the myenteric plexus of the ENS, as well as the number of nitrergic nerve fibers in the muscular layer of the descending colon. The obtained results suggest that NO is involved in the pathological condition of the large bowel and probably takes part in neuroprotective and/or adaptive processes.

## 1. Introduction

The enteric nervous system (ENS), located in the wall of the gastrointestinal (GI) tract controls the majority of functions of the stomach and intestine [1]. It is characterized by a significant number of neuronal cells, a complex structure, and a high degree of independence from the central nervous system [2,3,4]. The specific structure of the ENS depends on the given species. In large mammals, (including the domestic pig), the ENS interacts with the small and large intestine and consists of three types of the intramural ganglionated plexuses: the myenteric plexus (MP), located in the muscular layer of the intestinal wall; the outer submucous plexus (OSP), located in the submucosal layer, near the internal side of the circular muscular layer; and the inner submucous plexus (ISP), also located in the submucosal layer but close to the intestinal lumen [5]. Enteric neurons are very diverse in terms of their neurochemical characteristics, morphological properties, and functions [1,4]. Apart from acetylcholine, the main neuromediator of the ENS, enteric neurons may synthesize a wide range of other active substances which play varying roles in neurotransmission [6,7].

To date, tens of such neurochemical factors in enteric neurons have been described. Among them, nitric oxide (NO) deserves particular attention. It is a gaseous neurotransmitter and/or neuromodulator which is synthetized from L-arginine in a reaction catalyzed by enzymes of the nitric oxide synthase group [8]. The action of NO depends on neurotransmitters such as glutamate and aspartate, which bind NMDA receptors [9], the stimulation of which indirectly leads to the activation of nNOS, considered as its marker. The neuronal isoform of nitric oxide synthase (nNOS) is typical for the nervous system and is considered to be a marker of nitrergic neurons and nerve fibers [10]. NO is widely distributed in the central and peripheral nervous systems [11,12,13]. It has also been found in relatively large quantities in the enteric neurons of various mammal species, including humans [12,13,14,15,16].

In light of previous studies, it is known that NO in the GI tract is an important inhibitory factor and is involved in various processes in the stomach and intestine [17]. Among other roles, NO is involved in the regulation of the secretory activity of the stomach and intestine, mesenteric blood flow, and immune processes [17,18,19]. NO acts as a nonadrenergic-noncholinergic neurotransmitter (NANC) in the digestive system, participating in the physiological relaxation of the intestines during the digestive process. In addition, excessive amounts of NO released have the ability to kill neurons whose fibers come into contact with neurons exhibiting nitric oxide synthases (NOS) activity [9]. An important role of NO is the regulation of functional activity, the growth and death of certain types of immune and inflammatory cells. In the presence of the high concentrations produced by NOS-2, NO is oxidized to reactive nitric oxide species (RNOS), which are capable of modifying S-nitroso thiols to transform essential signaling molecules such as kinases and transcription factors. Another role of RNOS is to inhibit some of the significant enzymes of mitochondrial respiration resulting in depletion of ATP and cellular energy. These actions may explain the multiple effects of NO in regulating inflammation [20]. However, the main function of NO in the ENS pertains to inhibition of gastrointestinal smooth muscle activity [21,22,23].

Moreover, it is known that the number of nitrergic enteric neurons changes under the influence of both physiological and pathological stimuli [24,25,26]. This phenomenon is probably connected with neuroprotective and/or adaptive reactions within the GI tract, but many aspects of the participation of nitrergic enteric neurons in pathological processes are still not clear. PGP 9.5 is a nerve cell-specific protein that was discovered around 1980. This protein is localized both in neurons, neuroendocrine cells in vertebrates [27], and in other non-neuroendocrine tissues such as smooth muscle, distal renal tubules, Leydig cells, oocytes, melanocytes, epididymis, Merkel cells, and skin fibroblasts. PGP9.5 is expressed at about 50-fold higher concentration in the brain than in other tissues and contains about 1–5% of total brain soluble proteins and is therefore used as a marker of nerve cells [28,29,30,31].

Therefore, the aim of the present study is to investigate the influence of experimental chemically-induced inflammatory processes on the number of nitrergic neuronal cells in the myenteric plexus, which first of all takes part in the regulation of the intestinal motility, as well as in nerves within the muscular layer of the porcine descending colon. The obtained results will contribute to a better understanding of the ENS response to inflammatory process and the various roles of NO in this type of process.

## 2. Materials and Methods

This experimental study was conducted using 15 commercial sows (8-weeks-old, 20 kg body weight) divided into 3 groups (5 animals in each group), namely control animals (C group), without any treatment, “sham” operated pigs (C 1 group), and sows in which experimental inflammation was induced (C2 group). Experimental procedures during this research were carried out according to the resolution of the Local Ethical Committee (LEC) in Poland (Resolution LEC in Olsztyn, Poland, no. 90/2007 and no. 85/2008). After typical veterinary anesthesia, the sows of C2 group were subjected to a laparotomy, during which chemically-induced inflammation was induced according the manner described previously by Makowska and Gonkowski [32]. In short, the induction of inflammation consisted of the microinjections of 5–8 μL (injections with 80 μL of 10% formalin) into the colonic wall (in the place where nerves from the inferior mesenteric ganglia reach the intestinal wall). The pigs of group C1 were injected in the same manner with a saline solution. Injections for the animals of group C1 were made in the same fragment of the descending colon.

The sows were euthanized and the same fragments of the descending colon (previously injected with formalin or saline solution, after 5 days) from all pigs were collected. These fragments were subjected to the routine double-labeling immunofluorescence technique described previously by Makowska and Gonkowski [32]. With this technique, two types of primary antibodies were used, namely antibody against protein gene product 9.5 (PGP 9.5, pan-neuronal marker, mouse, working dilution 1:1000, catalog no. 7863–2004, Biogenesis Ltd., Poole, UK), and an antibody against nNOS (rabbit, working dilution 1:2000, catalog no. AB5380, Merck Millipore, Warsaw, Poland). Complexes of primary antibodies and appropriate antigens were visualized with mixtures of secondary antisera species-specific conjugated to Alexa Fluor, specifically Alexa Fluor 546 donkey anti-rabbit IgG and Alexa Fluor 488 donkey anti-mouse IgG (working dilution 1:1000, both sourced from Invitrogen, Carlsbad, CA, USA, Table A1). Typical control procedures for the specificity of antibodies were performed, such as the pre-absorption of primary antisera with appropriate antigens, as well as omission and replacement tests.

Additionally, the intestinal fragments were subjected to routine macroscopic and microscopic examination to detect the presence of the inflammatory changes.

The fragments of the descending colon were evaluated under an Olympus BX51 microscope equipped with epifluorescence and appropriate filter sets. For determination of the percentage of nitrergic neurons, at least 500 neuronal cells immunoreactive (IR) to PGP 9.5 were counted for the presence of nNOS. The number of PGP 9.5-positive neurons was recognized as 100; in turn, the determination of the density of nerve fibers IR to nNOS in the muscle wall was based on the count of such nerves in the microscopic field of observation (0.1 mm^2^). The number of nerve fibers was evaluated with four fragments of the descending colon per animal (with five fields per section). To prevent double counting of the same nerves and neurons, the microsections of the descending colon included in the study were located at least 250 μm apart. Statistical analysis was performed using a one-way ANOVA test (Statistica 9.1, StatSoft, Inc., Cracow, Poland) and differences were considered statistically significant at *p* ≤ 0.05. The data were collected and presented here as the mean ± SEM.

## 3. Results

**Scheme 1:**
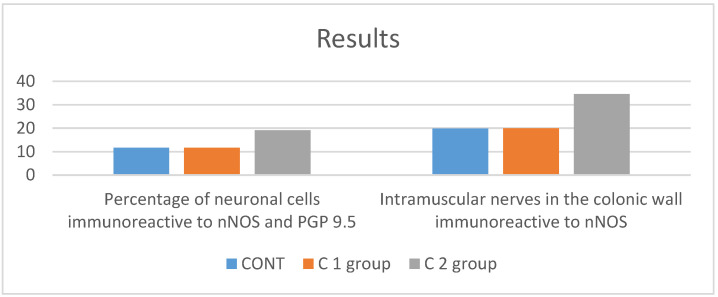
The percentage of neuronal cells nNOS/PGP 9.5 positive and the percentage of nitric oxide synthase (nNOS) immunoreactive fibers in the colonic wall in the control group (blue column), C1 group (orange column), and C2 group (grey column).

**Figure 1:**
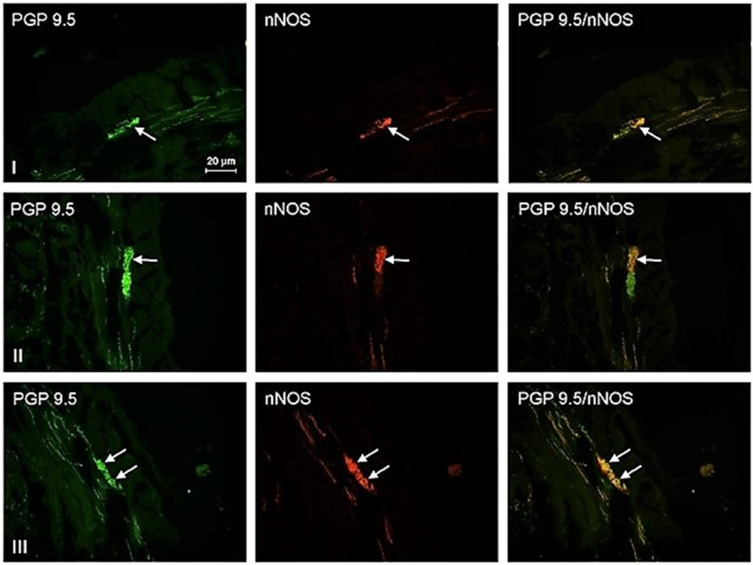
Myenteric neuronal cells in the descending colon immunoreactive to nNOS and PGP 9.5 (indicated with arrows) in the control (**I**), sham operated (**II**), and animals with chemically-induced inflammation (**III**).

During the present study, nervous structures that displayed immunoactivity to nNOS were observed in the muscular layer of the porcine descending colon, both under physiological conditions and during the inflammatory process (Figure 1). With the control animals, the percentage of neuronal cells immunoreactive to nNOS in the myenteric plexus amounted to 11.70 ± 0.94% of all myenteric neurons immunopositive to protein gene product 9.5. The number of nitrergic neurons observed in the majority of myenteric ganglia fluctuated from two to five neuronal cells. Only single ganglia did not contain nNOS-positive cells. In the “sham” group, the percentage of nNOS-positive neurons was 11.65 ± 0.56% and was not significantly statistically different from the values found with the experimental chemically-induced physiological condition. This fact suggests that manipulation on the intestine performed during the present study did not affect the number of nitrergic nervous structures. In turn, chemically-induced inflammation caused a clear increase in the percentage of myenteric neurons immunoreactive to nNOS. With the animals of group I, such neuronal cells amounted to 19.04 ± 1.09% of all PGP 9.5-positive cells. The majority of myenteric ganglia in the inflamed colon contained between four to eight neurons immunoreactive to nNOS (Scheme 1.)

**Figure 2:**
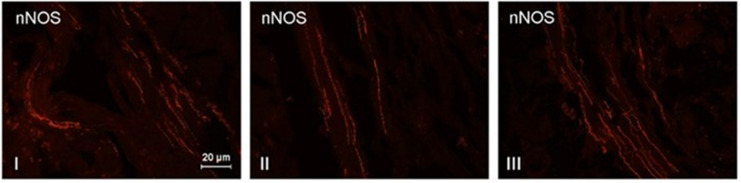
Intramuscular nerves in the colonic wall immunoreactive to nNOS (red) in the control animals (**I**), “sham” operated animals (**II**), and animals with inflammation (**III**).

Further visible changes were found for nitrergic nerves in the colonic muscular layer. Regarding the physiological conditions, the number of such fibers amounted to 19.80 ± 1.24 fibers per microscopic observation field for the control group. For the “sham” animals, the number was 19.96 ± 1.60 (there were no statistically significant differences between the C and C1 groups). For the animals subjected to inflammation, the number of such nerves was clearly higher and amounted to 34.60 ± 1.29 fibers per observation field. Moreover, the inflammation changed the morphology of nitrergic nerves. In the control group, the majority of nerves were delicate and thin, while with inflammation, nerves that contained nNOS were rather thick and easily visible (Figure 2).

## 4. Discussion

The relatively high number of nNOS-positive neuronal structures in the muscular layer of the pig descending colon observed during the present study confirms the important role of NO in the regulation of the intestinal motility, a concept which has been suspected in previous investigations [17,18,33].

Moreover, the changes examined during this research strongly suggest that NO takes part in mechanisms associated with chemically-induced inflammation. The increase in the number of nitrergic enteric neurons has also been shown with other types of inflammatory processes, induced both by microorganisms and toxic substances contained in food [23,34]. Studies on acrylamide-induced duodenitis in domestic swine show an increase in acrylamide-induced duodenitis in response to inflammation, and suggest that nNOS contributes to the protection of ENS neurons as an important line of defense against the damaging effects of acrylamide. However, on the other hand, the intensity of observed changes clearly depends on the type of inflammation, which suggests that the exact mechanisms leading to the increase in the numbers of nitrergic neurons are different between different inflammatory processes.

On the one hand, the increase in the number of nNOS-positive fibers and neurons may result from the pro-inflammatory activity of NO and its participation in the increase in pro-inflammatory cytokine levels [35,36]. It is also known that NO is a strong damaging factor. As a very active molecule, NO may react with a wide range of other intracellular factors, leading to blockage of important enzymes that produce free radicals. Such reactions, typically referred to as nitrative and/or oxidative stress, cause chemical changes in intracellular proteins, lipids, and nucleic acids [37].

On the other hand, it cannot be ignored that the changes noted in the present study may result from not only the damaging properties of NO, but also the protective properties. This thesis may be supported by previous studies, in which the participation of NO in neuroprotective processes has been described [38,39]. Moreover, it is known that NO is involved in enhancing neuronal resistance to damaging factors and the synthesis of protective substances in neuronal tissue [40,41].

Another reason for the changes noted in the present research may be connected with inflammation-induced disturbances of intestinal muscle activity. It is known that inflammatory processes stimulate the intestinal muscles, leading to diarrhea. Therefore, an increase in the synthesis of NO, which is known as a key factor for inhibiting intestinal motility [20,21,22], may be a manifestation of the adaptive processes aimed at the maintenance of homeostasis in the intestine. It is also possible that the increase in the number of nitrergic structures noted in this research is connected with inflammatory hyperemia, because NO is known as an important vasodilator, influencing mesenteric and intestinal blood flow [42]. Moreover, it cannot be ruled out that the increase in the nNOS-positive neuronal cells is connected with pain stimuli appearing during inflammation. This hypothesis is supported by the fact that nNOS has been described in neurons participating in sensation and pain stimuli conduction [43].

## 5. Conclusions

The present study has shown that a chemically-induced inflammatory process can increase the number of nNOS-positive neurons in the myenteric plexus (MP) of the porcine descending colon. The obtained results, on the one hand, show that the model of inflammation may be used as a simulation of other inflammatory processes in the intestine, and, on the other hand, demonstrate that nitrergic neurons observed in the myenteric plexus are involved in processes connected with inflammation. The observed changes may result from the participation of nitrergic neurons in adaptive processes in an inflamed intestine, but elucidation of the exact mechanisms of these fluctuations remains an objective for further studies.

## Data Availability

Not applicable.

## Abbreviations

ENS	enteric nervous system
NO	nitric oxide
GI	gastrointestinal
MP	myenteric plexus
OSP	outer submucous plexus
nNOS	neuronal isoform of nitric oxide synthase
PGP 9.5	protein gene product 9.5
IR	immunoreactive

## Appendix A

**Table A1:** Description of antibodies.

Antigen	Species of Origin	Code	Supplier	Country	Working Dilutions
**Primary antibodies**					
PGP-9.5	Mouse	7863-2004	Biogenesis	USA	1:1000
nNOS	Rabbit	AB5380	Merck Millipore	Poland	1:2000
**Secondary antibodies**					
Alexa fluor 488	Anti Mouse	A21206	Invitrogen	USA	1:1000
Alexa fluor 546	Anti Rabbit	A10036	Invitrogen	USA	1:1000
